# Editorial: Lipid droplets and mitochondria in metabolic diseases

**DOI:** 10.3389/fphys.2023.1266356

**Published:** 2023-08-11

**Authors:** Wen Su, Yujing Chi, Yu A. An

**Affiliations:** ^1^ Department of Pathophysiology, Shenzhen University Health Science Center, Shenzhen University, Shenzhen, China; ^2^ Department of Central Laboratory and Institute of Clinical Molecular Biology, Peking University People’s Hospital, Beijing, China; ^3^ Department of Gastroenterology, Peking University People’s Hospital, Beijing, China; ^4^ Department of Anesthesiology, Critical Care and Pain Medicine, McGovern Medical School, University of Texas Health Science Center at Houston, Houston, TX, United States

**Keywords:** lipid droplets, mitochondria–lipid droplet communication, non-alcoholic fatty liver disease, obesity, metabolic disease, mitophagy

The prevalence of metabolic diseases, including diabetes, obesity, and non-alcoholic fatty liver disease (NAFLD), has been increasing rapidly worldwide. Lipid droplet (LD) and mitochondria are metabolic hubs in oxidative tissues and organs. Growing evidence suggests that lipid droplets (LDs) and mitochondria are closely intertwined. The proximity of mitochondria to LDs has been widely observed by electron microscopy and fluorescence staining in metabolic active tissues such as the liver, brown adipose tissue, and muscle. Dynamic LD–mitochondria interactions enable the translocation of fatty acids derived from lipolysis of LD into mitochondria for fatty acid β-oxidation (FAO), in adaptation to various nutrient conditions. However, dysregulated LD metabolism can lead to impaired mitochondrial function, lipotoxicity, and related metabolic disorders, including obesity, insulin resistance, and NAFLD.

In this Research Topic of “*Lipid Droplets and Mitochondria in Metabolic Diseases*,” we have published six excellent articles and reviews that significantly advance the field.

Jia et al. investigated the role of mitochondria-targeted antioxidant SkQ1 in the pathophysiology of hemorrhagic shock. The authors provided compelling evidence that mitochondrial dysfunction plays a key role in the development of inflammation and oxidative stress following hemorrhagic shock. Huang et al. explored the role of CD34 expression in a rat model of type 1 diabetes (T1D). They demonstrated that CD34 is specifically expressed in rat islet α-cells and is negatively correlated with the islet β-cell number, demonstrating a novel promising biomarker for T1D. Ning et al. provided a detailed overview of the role of mitophagy in the development of insulin resistance (IR), which might be a potential target for therapies that improve IR and metabolic diseases. Yao et al. reported that the activation of AMPK can counteract fatty acid-induced LD accumulation, oxidative stress, and inflammatory response in primary chicken hepatocytes, showing a potential strategy for the prevention of fatty liver hemorrhagic syndrome in laying hens. Moreover, Meng et al. developed a sensitive mitochondrial thermometry 2.0, which is effective in the detection of mitochondrial thermogenic responses in brown adipocytes. Their study provides a useful tool in exploring the molecular basis of LD homeostasis and its relationship with mitochondrial function in live cells.

In addition to key proteins that modulate the lipid homeostasis and mitochondrial function, recent lipidomic studies reveal that certain lipid compositions, especially phospholipids, could affect LD morphology, the LD–mitochondria interaction, and mitochondrial function. Phosphatidylethanolamine (PE) and phosphatidylserine (PC) are two important types of phospholipids. Liu et al. provided a thorough summary of the current understanding of phosphoethanolamine/phosphocholine phosphatase 1 (PHOSPHO1) in regulating energy metabolism. The authors also highlighted the importance of the balance between PC and PE in determining ER homeostasis, mitochondrial function, and LD fusion and fission.

**FIGURE 1:**
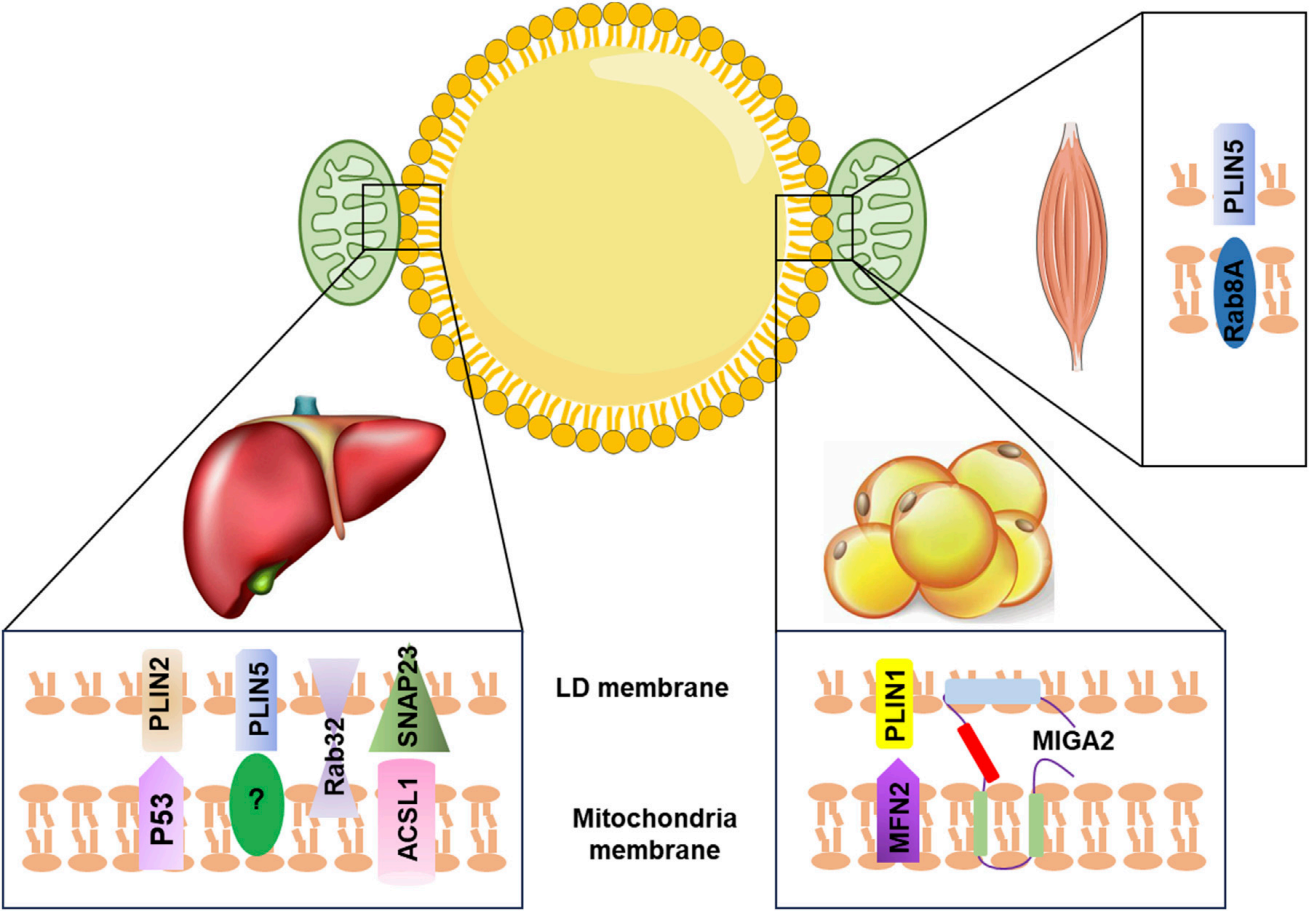
Summary of LD–mitochondria contact machinery in different tissues. In the liver, PLIN2 interacts with mitochondria p53 to promote LD–mitochondria contact. The binding partner for PLIN5 is currently unclear. Rab32 facilitates mitochondria–LD contact. SNAP23 interacts with ACSL1, which is located at the outer mitochondrial membrane to promote the tethering of mitochondria with LD. In adipose tissue, MIGA2, an outer mitochondrial membrane protein, directly tethers mitochondria with LD. PLIN1 interacts with MFN2 to promote mitochondria–LD contact. In the skeletal muscle, Rab8a acts as a mitochondrial receptor for LDs forming the tethering complex with the LD-associated PLIN5. LD, lipid droplet; PLIN2, perilipin 2; PLIN5, perilipin 5; P53, tumor protein p53; Rab32, Ras-related protein32; SNAP23, synaptosome-associated protein 23; ACSL1, acyl-CoA synthetase 1; MFN2, mitofusin 2; MIGA2, mitoguardin 2; PLIN1, perilipin 1; Rab8a, Ras-related protein 8A.

Recent studies have identified key proteins that play a role in the LD–mitochondria interaction. For instance, in the liver, exposure to aflatoxin B1 (AFB1) can cause liver injury through the interaction between mitochondrial p53 (mito-p53) and the LD-associated protein perilipin 2 (PLIN2), which impeded the lysosome-dependent lipophagy (Che et al., 2023). Overexpression of PLIN5 has been shown to promote LD formation and enhance LD–mitochondria contact, leading to reduced cellular ROS levels and upregulation of mitochondrial function-related genes, such as COX (cytochrome c oxidase subunit IV) and CS (citrate synthase), in HepG2 cells (Tan et al., 2019). Additionally, the interaction between synaptosome-associated protein 23 (SNAP23) and long-chain acyl-CoA synthetase 1 (ACSL1), located on the mitochondrial surface, can promote LD–mitochondria contact, which enhances the flow of fatty acids released from LDs into the mitochondrial oxidation machinery (Young et al., 2018). Other proteins, such as mitoguardin 2 (MIGA2), Ras-related protein 8A (Rab8a), and Ras-related protein32 (Rab32), have also been found to mediate LD–mitochondria contact and regulate triacylglycerol turnover, FA oxidation, and mitochondrial respiration (Stone et al., 2009; Freyre et al., 2019; Song et al., 2020; Ouyang et al., 2023) (Figure 1). Despite the importance of LD–mitochondria interaction, the exact mechanism of how these two organelles interact during metabolic diseases remains unclear and requires further investigation.

Whether the LD-associated mitochondria (LDM) facilitate a high FAO rate or low FAO rate is still under debate. Understanding the physical roles and regulation of mitochondrial tethering to lipid droplets and identifying key proteins are of substantial significance. Some barriers limit the full understanding of the role of LD–mitochondria interaction in metabolic diseases: first, the lack of a stable method to isolate LDM; second, a limited number of identified critical proteins that mediate the LD–mitochondria interaction; and third, the lack of specific probes for LDM.

In summary, given the crucial roles of LD–mitochondria contact in regulating lipid metabolism, it is necessary to further investigate how the loss or enhancement of these contact sites affects lipid synthesis and lipolysis. With the support of sufficient evidence, LD–mitochondria contact sites could potentially serve as a therapeutic target for metabolic diseases.
